# Evaluating the Safety of Thread-Embedding Acupuncture: Protocol for a Multi-Center, Prospective, Observational Study in Clinical Practice

**DOI:** 10.3390/healthcare13020135

**Published:** 2025-01-13

**Authors:** Seojung Ha, Changwoo Seon, Jinyeong Hong, Bonhyuk Goo, Eunseok Kim, Suji Lee, Myung-Sook Lyou, Ye Ji Shin, Jung-Hyun Kim, Yeonju Woo, Bo-In Kwon, Jin-woo Suh, Dong Hyuk Lee, Sang-Soo Nam, Joo-Hee Kim

**Affiliations:** 1 Department of Acupuncture and Moxibustion Medicine, College of Korean Medicine, Sangji University, Wonju-si 26339, Republic of Korea; sangjiacu2@sangji.ac.kr (S.H.);; 2 Department of Acupuncture & Moxibustion, Kyung Hee University Hospital at Gangdong, Seoul 05278, Republic of Korea; goobh@khnmc.or.kr (B.G.); dan-mi725@khnmc.or.kr (J.-H.K.); dangun1966@khu.ac.kr (S.-S.N.); 3 Department of Acupuncture and Moxibustion Medicine, Pusan National University Korean Medicine Hospital, Yangsan 50612, Republic of Korea; eskim@pusan.ac.kr; 4 Department of Acupuncture and Moxibustion Medicine, Kyung Hee University Medical Center, Seoul 02447, Republic of Korea; sjstarry41@naver.com; 5 SHE’S Korean Medicine Clinic, Seoul 06614, Republic of Korea; 6 Department of Physiology, College of Korean Medicine, Sangji University, Wonju-si 26339, Republic of Korea; justice@sangji.ac.kr; 7 Research Institute of Korean Medicine, Sangji University, Wonju-si 26339, Republic of Koreaneurohani_ldh@sangji.ac.kr (D.H.L.); 8 Department of Pathology, College of Korean Medicine, Sangji University, Wonju-si 26339, Republic of Korea; 9 Department of Korean Neuropsychiatry, College of Korean Medicine, Sangji University, Wonju-si 26339, Republic of Korea; 10 Department of Anatomy, College of Korean Medicine, Sangji University, Wonju-si 26339, Republic of Korea; 11 Department of Acupuncture & Moxibustion, Kyung Hee University College of Korean Medicine, Kyung Hee University Hospital at Gangdong, Seoul 05278, Republic of Korea

**Keywords:** thread embedding acupuncture, polydioxanone, safety, prospective, observational study

## Abstract

**Background/Objectives:** Medical therapies that apply biodegradable materials, such as polydioxanone, are widely used to treat various disorders. Thread-embedding acupuncture (TEA) is a unique form of acupuncture that exerts long-lasting therapeutic effects by inserting absorbable threads at specific acupuncture points, and is widely used to treat various diseases. However, there is currently a lack of research regarding the safety of TEA. This prospective observational trial aims to evaluate the safety of TEA by collecting and analyzing data related to adverse events in patients receiving TEA in actual practice. **Methods:** A total of 350 eligible participants who undergo TEA at one of three university-affiliated hospitals and two traditional Korean medicine clinics will be systemically observed for post-treatment adverse reactions. The patients will be monitored at three time points: 1 week, 1 month, and 3 months post-treatment. Safety evaluations will assess the incidence of adverse events and treatment discontinuation rates during the 3-month post-treatment period. **Conclusions:** This study will evaluate the safety of TEA and provide information for decision-making in clinical practice as well as basic data for future large-scale research.

## 1. Introduction

Medical therapies that apply biodegradable materials, most commonly polydioxanone, are being utilized to treat various disorders and medical conditions. Thread-embedding acupuncture (TEA) is a type of acupuncture that involves the insertion of absorbable threads into the subcutaneous tissue or muscles for extended therapeutic stimulation. This specialized thread not only provides mechanical support, but also induces a localized inflammatory response, aiding in tissue recovery [1,2].

TEA has been practiced for centuries, supported by extensive clinical experience and empirical evidence in traditional East Asian medicine [3,4]. Unlike many conventional treatments that undergo preclinical evaluations before clinical application, TEA and similar practices in traditional East Asian medicine have followed a different pathway. These treatments have often been integrated into clinical practice first, based on long-standing empirical use and observed clinical benefits [5]. Subsequently, they undergo modern evaluations to systematically assess their safety and efficacy [6]. This approach reflects the practical, experience-based nature of East Asian medicine, where treatments are continuously refined through extensive real-world use.

TEA is used for various conditions, including musculoskeletal disorders [7,8], neurological conditions [9,10], allergic rhinitis [11], abdominal obesity [12], nonalcoholic fatty liver disease [13], and gastroesophageal reflux disease [14]. It is also applied in cosmetic procedures to reduce wrinkles and enhance skin elasticity [15,16]. Given its diverse applications, the materials and protocols used in TEA can vary significantly. Threads such as polydioxanone (PDO), polycaprolactone (PCL), and polylactic acid-glycolic acid are used, each with different degradation rates and tissue responses [17,18,19,20].

Despite its widespread use, there is a lack of systematic safety evaluations for TEA. Given that TEA is an invasive procedure, it carries the risk of adverse events (AEs), especially when performed by unskilled providers or in unsafe conditions [21]. Potential AEs include foreign body granuloma formation [22,23], skin infections [24], dimpling [25], and parotid gland injuries [26]. A retrospective study investigating the clinical effectiveness and safety of TEA in patients with facial palsy sequelae reported that more than 200 TEA treatments were administered in the 82 patients included in the study, with no serious adverse events (SAEs) or AEs requiring medical treatment [27]. In addition, a review article reporting AEs of TEA in musculoskeletal conditions found that 22 studies reported AEs out of a total of 97 retrieved articles, with 10 studies reporting no AEs or AEs unrelated to TEA. The most common AEs reported were redness, stiffness, and bruising, most of which were mild and transient [28]. However, retrospective studies have several limitations, such as those involving missing information, and selection and recall bias, due to the nature of the study’s design [29].

In a network meta-analysis of systematically evaluating the effectiveness and safety of acupuncture-related therapies in patients with polycystic ovary syndrome, 39 RCTs comparing 14 acupuncture-related therapies involving more than 4600 patients with polycystic ovary syndrome were analyzed. TEA and medication were the most frequently used treatments, followed by moxibustion and herbal medicine, moxibustion alone, acupuncture and medication, acupuncture–moxibustion and auricular acupuncture, and acupuncture and auricular acupuncture [30]. Nevertheless, existing clinical literature primarily consists of case reports and small-scale studies focusing on specific AEs [23,31]. Therefore, there is an urgent need for a well-designed prospective protocol to systematically assess the safety of TEA in real-world clinical settings.

This multicenter, prospective, observational study aims to address this gap by evaluating the safety of TEA. Previous studies have primarily focused on traditional acupuncture, which has been reported as safe in several large-scale sample surveys [32]. However, TEA, being a unique and more invasive form of acupuncture, requires a more comprehensive evaluation of its safety profile. This study aims to examine AEs associated with TEA and explore how patient-, practitioner-, and intervention-related factors influence these events, ultimately contributing to a better understanding of TEA’s safe practice.

## 2. Methods

### 2.1. Study Design, Setting and Aim

**Figure 1:**
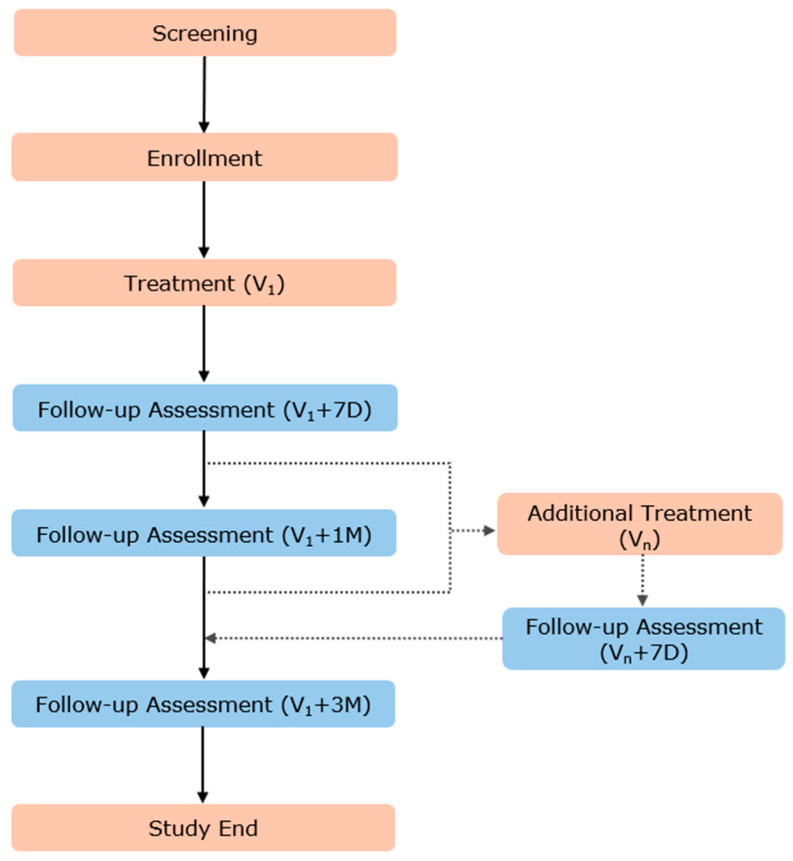
Flowchart of the observational study. Abbreviations 7D, 1M, and 3M represent 7 days, 1 month, and 3 months after TEA treatment, respectively.

This multicenter, prospective, observational study will evaluate the safety of TEA in clinical practice. The study protocol is registered with the Clinical Research Information Service (CRIS; https://cris.nih.go.kr, (accessed on 16 December 2024); registration number KCT0008912). Participants will be recruited from three university-affiliated hospitals and two traditional Korean medicine clinics in Korea. Patients receiving at least one TEA treatment in a real-world setting, in line with the eligibility criteria, will be enrolled, and all AEs occurring in participants for a total of 3 months will be prospectively collected. In clinical practice, each patient will receive an appropriate number of TEA treatments based on their disease and condition; therefore, all additional TEA treatments after the first treatment will be collected as V2 to Vn in this study. Data regarding all TEAs received by participants and all AEs occurring during the study period will be collected and evaluated. The study procedure and assessment protocol for this trial are presented in Figure 1 and Table 1.

This study aimed to prospectively evaluate and validate the safety of TEA in real-world clinical practice. This observational approach enables the collection of safety data from a diverse patient population to reflect the complexities of actual clinical practice.

### 2.2. Participants

#### 2.2.1. Eligibility Criteria

Adults ≥ 19 years old who are scheduled to undergo TEA at one of the participating Korean medical institutions during the study period will be included in this study. Participants will be excluded if they have a condition or circumstance that the investigator determines may interfere with their participation in the study, such as having a serious comorbidity or inability to comply with the study protocol. In addition, each patient must provide written informed consent, demonstrate a clear understanding of the study’s objectives, comprehend the follow-up visit requirements, and express willingness to provide necessary information during follow-up visits.

#### 2.2.2. Recruitment Process

Patients undergoing TEA will be recruited from three university-affiliated hospitals and two traditional Korean medicine clinics in Korea. Recruitment will be carried out through direct referrals from medical staff at these institutions, as well as through consultation with the research team during routine clinical visits. No recruitment will be conducted using public advertisements. A total of 350 patients will be recruited (See the sample size calculation section below). Written informed consent will be obtained before initiating any research-related procedures. Patients will have the opportunity to ask questions regarding the study and will be assured that their decision to participate or not will not impact their clinical care.

### 2.3. Interventions

#### TEA Procedure

Standard TEA procedures will be followed, involving the insertion of absorbable threads using guide needles. For each participant, detailed information regarding the TEA procedure will be recorded, including the target disease or reason for undergoing TEA, such as cosmetic purposes, lumbar disorders, cervical disorders, shoulder disorders, elbow disorders, wrist disorders, knee disorders, ankle disorders, facial nerve palsy, facial spasm/twitch, temporomandibular joint disorders, gastrointestinal disorders, urinary system disorders, and gynecological disorders. The specific TEA products and their manufacturers will be documented. The types of threads used will include various compositions such as PDO and PCL, and forms, such as mono, multi, screw, and cog, based on the number of threads, twists, and the presence or absence of barbs [33]. The treatment areas will be carefully noted, covering the facial region, cervical region, shoulder region, lumbar region, gluteal region, thoracic and abdominal regions, upper limbs, and lower limbs. The number of thread insertions in each area will also be documented. Thread sizes, ranging from 7-0 to 3-0, and needle lengths and thicknesses will be recorded. The depth of thread insertion will be documented, with threads being inserted subcutaneously, into the fascia, intramuscularly, or into the periosteum. Concurrent treatments, such as acupuncture, moxibustion, and herbal medicine, will be permitted and recorded during the trial.

### 2.4. Data Collection

#### 2.4.1. Baseline Data

Demographic information, vital signs, medical history, concomitant medication and treatment details will be collected for each participant at the start of the study. This includes patient factors (age, sex, height, weight, blood pressure, pulse, body temperature, medical history, AEs of TEA within one year, medication history within one week, and concomitant medications or treatments), practitioner background factors (length of practice, affiliations, and board certification), and intervention factors (target disease, product manufacturer, thread material, thread shape, thread size, treatment area, number of needle insertions, needle length, needle thickness, and needling depth).

#### 2.4.2. AE Monitoring

Definition of AEs [34]: AEs are defined as any unfavorable or unintended signs, symptoms, or diseases temporally associated with the TEA procedure.

Monitoring Schedule: Patients will be monitored at baseline (V1: enrollment period and first TEA treatment), 1 week post-treatment (V1 + 7D), 1 month post-treatment (V1 + 1M), 3 months post-treatment (V1 + 3M), and during intermediate procedures performed within 3 months post-treatment (evaluated 1 week later—Vn + 7D). In addition, participants will be encouraged to self-report AEs at any time during the study period using an application or diary. Symptom confirmation and evaluation will be conducted on the designated assessment dates.

All symptoms will be independently assessed by an investigator blinded to the specific TEA details and subsequently confirmed for AEs by the practitioner who performed the TEA to ensure that no AEs are omitted that are unrecognized by the patient or the blinded assessor. Patients will also be encouraged to voluntarily upload photos of AEs to the self-reporting application if they would prefer, so that they can be independently assessed by blinded researchers and medical staff. In addition, depending on the AE, appropriate medical examination and management, including laboratory tests and imaging, will be applied until the AE resolves.

#### 2.4.3. Outcome Measures

The development of a standardized AE-reporting form is essential to facilitate accurate data collection, improve the quality of post-medical treatment, and ensure patient safety. The Medical Dictionary for Regulatory Activities (MedDRA) is a standardized international lexicon that is used by regulatory authorities for data related to medical treatments [35]. In the present study, the safety information report was developed based on the integration of MedDRA system organ class (SOC) and preferred term (PT) and a Delphi, a well- structured technique using collective consensus of multiple clinical experts [36]. Symptoms that may be associated with TEA are presented in Table 2.

Causality is assessed by evaluating the following causal factors according to the WHO-UMC criteria [37], which are the most widely used worldwide.

- Is there a temporal relationship between the TEA and the occurrence of the AE?
- Does the same AE occur if TEA is repeated?
- Is it possible that the patient’s underlying medical condition or concomitant medication or procedure is the cause?
- Has the same AE been reported in the past?
- Did the AE improve after TEA was stopped?
- Is there a reasonable biological relationship between the procedure and the AE?

The outcome measure is the incidence, nature, and severity of AEs associated with TEA. During the 3-month post-treatment period, the incidence of AEs and rate of TEA treatment discontinuation due to AEs will be assessed. In addition, patient-, practitioner-, and intervention-related factors associated with the occurrence of AEs will be examined. The Standard Protocol Items: Recommendations for Interventional Trials (SPIRIT) schedule of enrollment, interventions, and assessments is shown in Table 1. The SPIRIT Checklist can be found in the Supplementary Files.

#### 2.4.4. Data Collection and Management

Trial staff will create a safety information report for each AE that occurs after TEA treatment. To ensure inter-rater standardization, trial staff were trained in a 1-day workshop on consistent assessment criteria, protocols, and reporting forms. The training included case scenarios, role-playing, and practical exercises, followed by performance assessments and feedback. Ongoing AEs at the time of the report will be verified at the next follow-up evaluation and documented until resolution. Patients will be encouraged to self-report AEs using an application. All data will be retained for three years after the study’s completion. For security, the source data will be protected using study identification numbers. Personal patient information will be stored in a subject identification log, which will be kept in a locked cabinet accessible only to authorized personnel. Protocol revisions will be managed by Sangji University Korean Medicine Hospital, the central coordinating facility. They will conduct site-level monitoring to ensure the protection of participant rights, and adherence to research protocols.

### 2.5. Statistical Analysis

#### 2.5.1. Sample Size Calculation

This prospective observational study will be conducted in a real-world clinical setting involving a diverse patient population with various diseases and clinical backgrounds. Given the expected high heterogeneity of the participants and high variance in the data, statistical sample size calculations are challenging. A previous study reported the incidence of AEs following TEA [38]; however, the data were based on published randomized controlled trials and case reports rather than actual clinical practice. To address these limitations, the sample size for this study was conservatively estimated at 12%, based on preliminary pilot data (unpublished). Using a margin of error of ±3.5% and a confidence level of 95%, the sample size calculation was performed. Accounting for an expected 5% dropout rate, the study aims to recruit approximately 350 participants.

$$n^{*}=\mathrm{pq}\frac{{\left(Z_{\alpha /2}\right)}^{2}}{d^{2}}=0.12\times (1-0.12)\times \frac{1.96^{2}}{0.035^{2}}=331.2,\;n=\frac{n^{*}}{\left(1-\omega \right)}=\frac{331.2}{0.95}\approx 349$$

#### 2.5.2. Statistical Methods

The analysis group will include participants who have received at least one TEA treatment. The main observation indicators, including the incidence rate of AEs and the treatment discontinuation rate, will be collected and analyzed without replacing the missing data. Continuous data will be represented using descriptive statistics, such as the number of participants, mean, and standard deviation, whereas categorical data will be presented as frequencies (N) or ratios (%). When necessary, a 95% confidence interval will be calculated. Unless otherwise specified, the statistical tests will be conducted using a two-sided significance level of 5%.

Student’s *t*-test will be used to compare normally distributed continuous variables and the Wilcoxon rank-sum test or Mann–Whitney U test will be used to compare non-parametric continuous variables between the patient groups with AEs and those without. A Shapiro–Wilk test and Kolmogorov–Smirnov test will be used to assess the normality of the distribution. The chi-square test (or Fisher’s exact test, when appropriate) will be used to compare dichotomous variables such as sex, the presence of comorbidities, and co-medications between the patient groups with and without AEs. Variables with *p* < 0.05 in the bivariate analysis or those considered clinically relevant will be included in the final regression model. The Kaplan–Meier method will be used to analyze the incidence of AEs and Cox proportional hazards regression will be used to analyze the differences in the incidence of AEs between subgroups including demographic characteristics, TEA materials or procedures.

All AEs collected in the study will be categorized according to the SOC and PT as defined in the MedDRA. The incidence and incidence rates will be presented by PT; however, multiple occurrences of the same AE by PT in the same subject will be considered as a single event. Furthermore, different discovered severities and causalities for the same AE will be treated as the maximum severities and causalities, respectively. Moreover, AEs associated with TEA and SAEs will be presented as events.

### 2.6. Ethics and Dissemination Plans

The study protocol declares that participants will be protected against invasion of privacy. This study has been approved by the Institutional Review Board of Kyung Hee University Korean Medicine Hospital at Gangdong (KHNMCOH 2023-06-002), Pusan National University Korean Medicine Hospital (PNUKHIRB 2023-06-001-001), Kyung Hee University Korean Medicine Hospital (KOMCIRB 2023-06-002-002), SHE’S Korean Medicine Clinic at Gangnam (KHNMCOH 2023-09-004), and SHE’S Korean Medicine Clinic at Sinchon (KHNMCOH 2023-09-005). This study will be conducted with respect for the individual participants according to the Declaration of Helsinki and Ethical Guidelines for Korea Good Clinical Practice [39]. The results of this study will be presented following data collection, published in a peer-reviewed journal, and reported at relevant conferences.

## 3. Discussion

TEA is a type of acupuncture that involves the insertion of absorbable threads into specific acupoints to produce enduring therapeutic effects. Previous studies have largely focused on its efficacy in treating various conditions, including musculoskeletal disorders, its use for cosmetic purposes, and for certain neurological conditions [27,40,41,42,43,44]. Some case reports and literature reviews have highlighted the need for comprehensive safety evaluations. For example, one study analyzed the safety of TEA in 61 clinical studies conducted in China and Korea and identified 28 types of AEs. Local reactions occurred in 9.83% (480/4882) of patients and systemic reactions occurred in 1.27% (62/4882) of patients. All five cases of SAEs (three cases of necrosis, one case of a skin ulcer, and one case of suppuration) occurred in patients in whom catgut thread was used [38]. Despite the increasing demand for TEA and several randomized controlled trials assessing its effectiveness and safety, there is a notable lack of systematic investigations into the AEs associated with TEA in real-world clinical settings.

By enrolling a large and diverse patient population across multiple centers, we aim to capture a comprehensive range of AEs and their potential correlations with different treatment parameters. Detailed documentation of each TEA procedure, including the type of threads, insertion techniques, and treatment areas, allows for a thorough analysis of factors that might influence the occurrence of AEs. Additionally, this study aims to evaluate safety with follow-up periods extending up to 3 months post-treatment. In our preliminary pilot study, most AEs occurred within the first 14 days, most often on the day of the procedure, and resolved spontaneously without any medical treatment. Furthermore, no additional AEs were observed after 3 months, suggesting that AEs were concentrated early on [45]. In addition, previous systematic reviews have reported that AEs following TEA were predominantly local reactions, most of which were mild, transient, and occurred in the acute post-treatment period [28,38]. This approach allows for the capture of both immediate and delayed AEs, contributing to a more comprehensive safety profile of TEA. Our systematic approach to data collection and analysis aims to contribute to improving evaluations in traditional East Asian medical practices. The systematic recording of treatment details and AEs can be applied to acupuncture, moxibustion, and herbal medicine studies.

For practitioners, understanding the risk profiles associated with different TEA techniques and materials can guide safer practice and improve patient outcomes. For patients, transparent safety information can help with making informed decisions regarding their treatment options. For researchers, the data will provide a foundation for further investigations into the mechanisms underlying AEs and the development of strategies to mitigate risks. Additionally, regulatory bodies and policymakers can use the findings to establish guidelines and standards for the safe practice of TEA and similar other interventions.

In conclusion, this study aims to contribute to the body of knowledge regarding the safety of TEA by providing systematic and comprehensive safety data. The data generated in this trial will serve as a foundation for further investigation and the development of evidence-based clinical guidelines.

### Strengths and Limitations

- This study is the first to systematically evaluate the safety of TEA in real-world clinical settings across multiple centers.
- Detailed documentation of the TEA procedure, including regarding the target disease, TEA products and manufacturers, thread types and specifications, treatment areas, and insertion techniques, allows for a comprehensive analysis of potential risk factors.
- The observational study design enables the capture of both immediate and delayed AEs over a 3-month follow-up period.
- To minimize recall and reporting bias, a mobile application has been developed to enable patients to self-report AEs at their convenience, which will be independently assessed by blinded assessors and double-checked by the practitioner.
- As an observational study, it is not possible to definitively establish causality between TEA and AEs.
- This study is conducted in a single country, which limits its applicability in other countries; however, it includes a diverse range of healthcare organizations and patient populations to enhance the generalizability of the findings.
- Inherent observer bias may affect the interpretation of the results of this study, and we aimed to minimize this bias by implementing inter-rater standardization in this study.

## 4. Conclusions

This study aims to provide a comprehensive safety profile of TEA by systematically evaluating AEs in real-world clinical settings. Our findings will provide valuable data on the safety of TEA, contributing to evidence-based clinical decision-making and the development of guidelines for safer practice. Although this study is limited by its observational nature and single-country setting, its detailed documentation of treatment parameters and systematic approach to data collection will serve as a foundation for future research.

## Figures and Tables

**Table 1:** Schedule of observations. The ● indicates at which point of the trial the respective assessments will take place. Vn represents additional treatments administered within the 3-month observation period. In such cases, follow-up assessments are conducted 7 days after each additional treatment to monitor any adverse events. Continuous self-reporting means that during the 3-month follow-up period, patients are encouraged to report any adverse events at any time using an app or diary.

	Screening	Registration		Observation Period				
Time Point	Screening Visit (V0)	Baseline (V1)	Additional Treatments (Vn)	One Week Post-Treatment (V1+7D)	One Week Post-Additional Treatment (Vn+7D)	One Month Post-Treatment (V1+1M)	Three Months Post-Treatment (V1+3M)	Continuous Self-Reporting
**Basic Condition**								
Informed consent	●							
Demographic information	●							
Vital signs	●	●	●					
Medical history	●							
Concomitant medication	●							
Inclusion/exclusion criteria	●							
**Confirmation of Schedule**								
TEA treatment		●	●	●	●	●	●	
Patient evaluation		●	●	●	●	●	●	
Physician evaluation		●	●	●	●	●	●	
**Evaluation of TEA Treatment Details**								
Target disease		●	●					
Product manufacturer		●	●					
Thread type		●	●					
Treatment area		●	●					
Number of insertions		●	●					
Thread specs		●	●					
Needle length/thickness		●	●					
Needle insertion depth		●	●					
Concurrent treatments		●	●					
**Safety Assessment**								
Incidence of adverse events			●	●	●	●	●	●
Symptoms			●	●	●	●	●	●
Date of onset			●	●	●	●	●	●
Date of resolution			●	●	●	●	●	●
Severity			●	●	●	●	●	
Causality			●	●	●	●	●	
**Others**								
Self-reporting education (app or diary)		●						
Changes in medical history			●	●	●	●	●	
Changes in concomitant medication			●	●	●	●	●	

**Table 2:** Symptoms that may be associated with TEA.

Type	Symptoms
Local symptoms	pain, sensation of foreign body, hemorrhage, bruising/hematoma, discoloration, redness, edema, abscess, dimpling (atrophy), myokymia (involuntary, localized muscle contractions), stiffness/muscle rigidity, induration/nodule/fibrosis, paresthesia, pruritus, nerve injury, salivary gland injury, and others
Systemic symptoms	pyrexia, chills, fatigue, pain, infection, dizziness, shock, angioedema, and others

## Data Availability

The primary investigator will have access to the final trial data set, which will be made available upon request.

## Supplementary Materials

The following supporting information can be downloaded at https://www.mdpi.com/article/10.3390/healthcare13020135/s1, The SPIRIT Checklist.
